# New Inroads Into Our Understanding of the Tauopathies, Alzheimer's Disease, and the Contribution of Altered Protein Conformation to Human Neurological Disease

**DOI:** 10.3389/fnins.2021.817983

**Published:** 2022-01-07

**Authors:** Walter J. Lukiw

**Affiliations:** Departments of Neurology, Neuroscience and Ophthalmology, LSU Health Sciences Center New Orleans, Louisiana State University, New Orleans, LA, United States

**Keywords:** Alzheimer's disease (AD), prion disease (PrD), protein folding, tauopathies, neurodegeneration

A substantial scientific advance has recently been made in the elucidation of the molecular mechanism by which unusually folded, self-associating tau proteins drive the neuropathology of several common types of human neurodegenerative disease. This group consists of about ~25 different, invariably lethal age-related tauopathies that include prominently, Alzheimer's disease (AD), argyrophilic grain disease (AGD), chronic traumatic encephalopathy (CTE), corticobasal degeneration (CBD), Parkinson's disease (PD), Pick's disease (PiD), and progressive supranuclear palsy (PSP) as well as familial British dementia, familial Danish dementia, primary age-related tauopathy, and several other more rare tauopathies. Normally neuron-abundant microtubule-associated protein tau (MAPT) proteins are involved in the organization and stabilization of the internal microtubule system of brain cells and are involved in the maintenance of overall neuronal shape, cytoarchitecture, and synaptic organization. Each of the tauopathies are defined by the progressive accumulation in the brain of MAPT proteins as complex, multi-component fibrillar aggregates, and their incidence and prevalence correlate strongly with the degree of dementia (Shi et al., 2021; Vaquer-Alicea et al., 2021). Interestingly, dominant tau mutations cause both increased tau aggregation, neuro-inflammation and neurodegeneration, and the pathogenesis of different tauopathies appear to involve pathological tau conformations that serve as templates that recruit native tau proteins to form abnormally folded tau proteins that support the generation of self-aggregating assemblies. These pathogenic molecular mechanisms: (i) may be augmented by other neurotoxic pathological factors that alter polypeptide conformation and promote protein misfolding; (ii) are extraordinarily similar to those involving the generation, propagation, diffusion, and spreading of prions; and (iii) have implications for the potential “seeding” and horizontal transmission of the entire spectrum of human misfolded tau-, α-synuclein- and amyloid-beta (Aβ) peptide-linked diseases that include AD, PSP and PD (Jaunmuktane and Brandner, 2020; Carlson and Prusiner, 2021; Thomzig et al., 2021).

Utilizing cryo-electron microscopy Shi et al. (2021) have recently formulated the basic principles for a hierarchical classification of tauopathies that can be made on the basis and nature of the unusual folded structuring of tau filaments in each disease type. More specifically, of the six tau isoforms naturally expressed in the human brain, three of these have 3 microtubule binding repeats (MTRs) and three have 4 MTRs (Falcon et al., 2018; Shi et al., 2021). Tauopathies can be classified into several basic groups based on the isoforms that constitute abnormal tau filaments, for example AD, CTE, PiD, and CBD are each characterized by different tau 3MTR- or 4MTR-type molecular folds (Falcon et al., 2018; Carlson and Prusiner, 2021; Vaquer-Alicea et al., 2021). Because of the unique and characteristic topology and topography of the 3MTR- and/or 4MTR-folded structures it should be possible to create a series of highly specific antibodies that discern between these various kinds of molecular folds, and hence the different types of tauopathies which to date have been exceedingly difficult to assess, categorize, and diagnose clinically. This important classification system should advance both diagnostic and prognostic methods for the early detection of neurodegeneration that will facilitate preclinical trials for experimental pharmaceuticals and also aid in the clinical management of these progressive prion-like diseases which currently have neither any effective treatment nor cure.

This systemic classification has already proved particularly useful in expanding our understanding of both the diversity in the clinical onset and presentation of tau-associated disease and the close molecular and structural interrelationships amongst different human tauopathies. One major challenge is to now develop analytical methodologies to accurately categorize patients according to the molecular structure of the underlying pathological tau protein assemblies to achieve more accurate diagnosis, prognosis, and effective clinical therapies for the wide spectrum of brain diseases involving misfolded tau and other neuronal-enriched fibrillar proteins such as α-synuclein (Carlson and Prusiner, 2021; Thomzig et al., 2021). These cryo-EM-based observations of misfolded tau proteins are also supportive of the prion hypothesis, which predicts that abnormal folding of endogenous natural protein structures into unusual pathogenic isoforms may over time contribute to and/or induce the atypical folding of other similar brain-abundant pathological proteins, while underscoring the age-related and progressive nature of these invariably fatal neurological disorders.

## Author Contributions

WJL reviewed the current literature in this area of neurodegenerative disease research and wrote this Opinion article.

## Funding

This research was supported through an unrestricted grant to the LSU Eye Center from Research to Prevent Blindness (RPB), The Brown Foundation, Joe and Dorothy Dorsett Innovation in Science Healthy Aging Award; the Louisiana Biotechnology Research Network (LBRN) and NIH grants NEI EY006311, NIA AG18031, and NIA AG038834 (WJL).

## Conflict of Interest

The author declares that the research was conducted in the absence of any commercial or financial relationships that could be construed as a potential conflict of interest.

## Publisher's Note

All claims expressed in this article are solely those of the authors and do not necessarily represent those of their affiliated organizations, or those of the publisher, the editors and the reviewers. Any product that may be evaluated in this article, or claim that may be made by its manufacturer, is not guaranteed or endorsed by the publisher.
